# Antimicrobial prescribing practices at a tertiary-care center in patients diagnosed with COVID-19 across the continuum of care

**DOI:** 10.1017/ice.2020.370

**Published:** 2020-07-24

**Authors:** Ryan W. Stevens, Kelsey Jensen, John C. O’Horo, Aditya Shah

**Affiliations:** 1 Department of Pharmacy Services, Mayo Clinic, Rochester, Minnesota; 2 Department of Pharmacy Services, Mayo Clinic Health System, Austin, Minnesota; 3 Division of Pulmonary and Critical Care Medicine, Mayo Clinic, Rochester, Minnesota; 4 Division of Infectious Disease, Mayo Clinic, Rochester, Minnesota

## Abstract

In a single-center review of antibiotic prescribing in COVID-19 patients, 10% of patients received antimicrobials, and inpatients encounters had the highest rate and spectrum of prescribing. Prescribing rate, spectrum, and duration appeared to increase with disease severity in inpatients. Antimicrobial prescribing in patients managed in ambulatory encounters was less common.

Detailed data surrounding antimicrobial usage in patients with coronavirus disease 2019 (COVID-19) are lacking. To date, our institution has not been an epicenter of the pandemic. Thus, the slow but consistent flow of cases has allowed for careful consideration of each case for candidacy for clinical trial enrollment, and formal infectious diseases consultation is provided for all inpatient encounters. Despite low local prevalence at the time, we found a cursory review of antibiotic prescribing early in the pandemic to be imperative, setting the stage for adjustment of antimicrobial stewardship practices if a future surge is to be observed. As we enter further into the realm of the unknown, data continue to emerge outlining indirect outcomes of COVID-19. One such outcome is the rate of coinfection with other pathogens and the associated use of antimicrobials. (1) Currently, data are sparse concerning antimicrobial management strategies for COVID-19. One review posited that coinfection during COVID-19 is rare; however, antimicrobial use among inpatients is common. (2) Some organizations have published guidelines to help streamline antimicrobial use in patients with pneumonia during the COVID-19 pandemic. (3) Despite the acute COVID-19 pandemic, antimicrobial resistance continues to be an ongoing and silent epidemic, accounting for ~3 million cases and ~35,000 deaths annually. (4) As the pandemic evolves, consideration should be given to evaluating the impact of potential excessive and/or unnecessary antimicrobial use in COVID-19 patients to mitigate unintended consequences, including adverse effects, unnecessary cost, and antimicrobial resistance.

## Methods

We retrospectively evaluated local patients diagnosed with COVID-19 at our large, academic, tertiary-care center during an inpatient, emergency department (ED), or outpatient encounter to analyze the prevalence, number of agents, spectrum of activity, and duration of antimicrobial therapy. We included patients ≥18 years of age with a confirmed case of COVID-19 (ie, a positive real-time polymerase chain reaction [RT-PCR] assay for severe acute respiratory coronavirus virus 2 [SARS-CoV-2]) diagnosed locally between March 1 and April 28, 2020. Only local patients were included to maintain uniformity of practice and review and to better understand local prescribing practices. Patients were excluded if they had not given consent for data utilization through the Minnesota research authorization. Patients were identified with a report of positive RT-PCR generated from the electronic health record. Patients were assigned to groups according to the encounter type during which they were diagnosed with COVID-19 (ie, inpatient, ED, or outpatient).

Given a significant mismatch in patient distribution across encounter types, we opted to describe our findings with descriptive statistics. From individual chart reviews, we evaluated patient demographics, risk factors, prevalence and result of other diagnostic testing (ie, respiratory cultures, respiratory virus pathogen panels [RVPs], or influenza testing), and disease severity. Mild disease was defined as no additional oxygen requirement, moderate disease as new or increased need for supplemental oxygen, and severe disease as need for invasive or noninvasive ventilation or intensive care unit (ICU) admission. Risk factors for outpatients and inpatients were obtained from the problem list and inpatient notes, respectively.

Antimicrobial prescribing data were included when employed for a suspected or confirmed respiratory tract infection. Antimicrobial utilization was evaluated by overall prescribing rate, days of therapy (DOT), DOT per antimicrobial day, and duration of therapy. Inpatients discharged on oral antimicrobials were included in overall antimicrobial metrics. For patients managed in ED or outpatient encounters, antimicrobials prescribed for a respiratory indication within 14 days of the testing date were reported. To provide assessment of the antimicrobial spectrum across various encounter types, an antimicrobial spectrum score developed by Gerber et al^5^ was applied to antimicrobial use data. Total spectrum scores were calculated and normalized by duration of therapy to determine the antimicrobial spectrum score per total antimicrobial day.

**Table 1.** Population Demographics

Characteristics	Full Cohort (n = 346), %^a^	Inpatient, %^a^				Ambulatory, %^a^		
		All (n = 39)	Mild (n = 7)	Moderate (n = 17)	Severe (n = 15)	All (n = 307)	Emergency Department (n = 20)	Outpatient Encounter (n = 287)
Age, mean y ±SD	45 ± 18	59 ± 19	42 ± 16	66 ± 14	58 ± 20	44 ± 17	51 ± 19	43 ± 17
Male	49	46	29	47	53	49.5	55	49
Mean Charlson comorbidity index ± SD	2 ± 3	4 ± 4	3 ± 4	4 ± 4	4 ± 3	2 ± 3	2 ± 3	2 ± 3
Length of stay, mean d ±SD	N/A	8.5 ± 5	2.6 ± 0.7	9.1 ± 4.7	11.5 ± 3.5	N/A	N/A	N/A
**Risk factors**								
Nursing home bound	2	3	0	0	7	2	10	2
Diabetes	15	31	14	41	27	2	10	13
Hypertension	18	23	0	35	20	18	30	17
Heart failure	3	13	0	24	7	2	15	1
Hyperlipidemia	16	18	0	29	13	15	10	16
Obesity	21	36	14	35	47	19	10	19
Malignancy	3	15	14	12	20	2	5	2
Lung disease	8	18	0	24	20	7	0	8
Age >65 y	10	36	14	53	27	7	25	6
CKD	3	13	14	18	7	2	5	2
Immunocompromising condition	3	5	0	6	7	3	0	3
No risk factors	53	23	71	12	12	57	50	57
1–2 risk factors	30	33	14	24	53	30	25	30
3–5 risk factors	15	38	14	53	33	12	25	11
>5 risk factors	2	5	0	12	0	1	0	1

Note. N/A, not available; SD, standard deviation; CKD, chronic kidney disease.

a Units unless otherwise specified.

**Table 2.** Antimicrobial Utilization

Antimicrobial Metric	Full Cohort (n = 346)	Inpatient				Ambulatory		
		All (n = 39)	Mild (n = 7)	Moderate (n = 17)	Severe (n = 15)	All (n = 307)	Emergency Department (n = 20)	Outpatient Encounter (n = 287)
Antibiotics Rx, no. (%)	33 (10)	23 (59)	2 (29)	8 (47)	13 (87)	10 (3)	3 (15)	7 (2)
Duration, mean d ±SD	5.7 ± 2.7	5.4 ± 2.9	5.5 ± 4.5	3.5 ± 2.7	6.5 ± 2.3	6.3 ± 2.1	6.8 ± 2.0	6.0 ± 2.0
Mean spectrum score per antimicrobial day ± SD	8.0 ± 3.5	9.2 ± 3.2	8.1 ± 7.0	8.4 ± 1.5	9.9 ± 2.7	4.9 ± 2.0	5.5 ± 2.3	4.4 ± 1.6
Total DOT/antimicrobial days	1.59	1.65	1.18	1.29	1.84	1.35	1.19	1.5
Total DOT	287	205	13	36	156	77	32	45
Ceftriaxone DOT	53	53	0	12	41	0	0	0
Cefdinir DOT	20	0	0	0	0	20	10	10
Cefazolin DOT	2	2	2	0	0	0	0	0
Azithromycin DOT	74	44	1	13	30	30	10	20
Doxycycline DOT	15	10	0	1	9	5	0	5
Vancomycin DOT	26	26	2	2	22	0	0	0
Piperacillin/Tazobactam DOT	28	28	0	8	20	0	0	0
Cefepime DOT	31	31	1	0	30	0	0	0
Metronidazole DOT	2	2	0	0	2	0	0	0
Amoxicillin DOT	5	0	0	0	0	5	5	0
Amoxicillin/Clavulanate DOT	0	0	0	0	0	0	0	0
Penicillin DOT	10	0	0	0	0	10	0	10
Cefuroxime DOT	2	2	0	0	2	0	0	0
Cefadroxil DOT	7	7	7	0	0	0	0	0
Levofloxacin DOT	12	5	0	5	0	7	7	0

Note. SD, standard deviation; Rx, prescription; DOT, days of therapy.

## Results

In total, 346 patients met inclusion criteria (Table 1). The mean age of the total population was 45 years, and 49% were men. Inpatient encounters accounted for 39 patients (11.3%): 7 cases (17.9%) were mild, 17 cases (43.6%) were moderate, and 15 cases (38.5%) were severe. Also, 4 inpatients remained admitted at the time of article submission, with data collected through May 5, 2020. Ambulatory encounters accounted for 307 patients, with 20 (5.8%) diagnosed in the ED and 287 (82.9%) diagnosed as outpatients. All patients diagnosed as outpatients were diagnosed by collection of specimens in a drive-through testing station with follow-up by virtual or telephone encounters. Of all patients initially managed in the ambulatory setting, 25 (8%) had follow-up ED encounters and 12 (4%) were admitted as inpatients before May 5, 2020.

Respiratory cultures were collected from 8 patients, all in the inpatient cohort: 6 were collected by tracheal aspiration and 2 by expectorated sputum. A causative pathogen (methicillin-sensitive *Staphylococcus aureus*) was only identified in 1 culture. Respiratory viral panels were collected in 4 patients and from the entire inpatient cohort, none of which identified a coinfecting pathogen. Influenza testing was performed in 12 inpatients (31%), 2 outpatients (1%), and no patients in the ED cohort. All influenza tests were negative.

Across the entire cohort, 10% of patients received antimicrobial therapy for a mean duration of 5.7 days, with a mean spectrum score per DOT of 8 (Table 2). Antimicrobials were administered in 59% of all inpatients with rates of 29%, 47%, and 87% for mild, moderate, and severe cases, respectively. The average number of DOT per antimicrobial day in all inpatients was 1.65: 1.18 for mild cases, 1.29 for moderate cases, and 1.84 for severe cases, respectively. The mean spectrum score per day of antimicrobial therapy was 9.2 in all inpatients and increased with disease severity, with mean scores per day of therapy of 8.1, 8.4, and 9.9 in mild, moderate, and severe cases, respectively. The mean duration of therapy in inpatients was 5.4 days: 5.5 days for mild cases, 3.5 days for moderate cases, and 6.5 days for severe cases.

Antimicrobial prescribing rates were considerably lower for ambulatory patients (3% overall), with ED and outpatient encounters demonstrating prescribing rates of 15% and 2%, respectively. Ambulatory antimicrobials were prescribed for a mean duration of 6.3 days: 6.8 days for ED encounters and 6 days for outpatient encounters. Ambulatory regimens generally contained fewer antimicrobials (1.35 DOT per antimicrobial day) and appeared to have narrower spectra (mean spectrum score per day of antimicrobial therapy, 4.9) than inpatient regimens. Across all included patients, no prescriptions for oseltamivir were issued.

## Discussion

During the COVID-19 pandemic, a reasonable amount of apprehension from providers regarding withholding antibiotics is expected, and vigilance by antimicrobial stewardship teams to identify and intervene upon unnecessary or inappropriate use is essential for mitigation of their unintended adverse consequences. The overall incidence of bacterial coinfection in patients with COVID-19 seems to be ~10%, based on various studies emerging from China, Italy, and the United States.^6–12^ Despite this fact, the overall rate of antimicrobial usage, especially among inpatients, appears to be high. A review of 18 studies reporting antimicrobial use in patients with COVID-19 found the prescribing rate to be 72%, with no antimicrobial stewardship interventions described in these studies to combat overuse.^2^ Data demonstrating clinical benefit of antibiotics as part of the routine management of patients with COVID-19 are lacking; retrospective cohort studies report similar or higher rates of antibiotic use in COVID-19 nonsurvivors compared to survivors.^13,14^

With concerns being raised for a wave of antimicrobial resistance following this pandemic, we sought to evaluate our early inpatient prescribing data to determine whether it correlated with other available reports. We identified similar rates of antimicrobial use in inpatients as seen in other recent studies, and, of unique value, we identified that very few outpatients received antimicrobials when diagnosed with COVID-19. We also found that coinfections with other pathogens were infrequent. Overall, antimicrobials were prescribed in 10% of patients. Inpatient admissions led to a higher prescription rates (59%) compared to ambulatory encounters (3%). We applied a previously described spectrum score to demonstrate the spectrum of antimicrobials used. This score assigns each antimicrobial a numerical score based on its spectrum of activity.^5^ In our review, when antimicrobials were prescribed, the duration of therapy appeared similar between inpatient and ambulatory encounters, but inpatients tended to receive broader-spectrum agents, as demonstrated by the mean spectrum score per day of antimicrobial therapy (9.2 for inpatients vs 4.9 for outpatients). Among the inpatient cohort, patients with severe disease received the broadest spectrum agents and longest durations of antimicrobials. Among the outpatient cohort, in addition to the relative infrequency of outpatient antimicrobial prescribing, narrower-spectrum antimicrobials were used in comparison to inpatient regimens.

Although comparative data pertaining to outpatient antimicrobial prescribing in COVID-19 patients are not available, our outpatient results represent an important finding, given increased attention to ambulatory antimicrobial stewardship with the new Joint Commission regulatory standards in 2020. Aside from COVID-19, other studies have evaluated antimicrobial prescribing patterns in outpatient settings for respiratory indications unlikely to benefit from antimicrobials and found prescribing rates of ~21%.^15,16^ Factors that influence inappropriate prescribing of antimicrobials include diagnostic uncertainty, time pressures, and perception of patient pressure antimicrobial prescribing.^17^ A potential hypothesis for our low rate of antimicrobial prescribing in the outpatient setting is that ambulatory encounters involved cases that were milder, for which the positive RT-PCR provided a level of diagnostic certainty, and for which time-related and patient-driven antibiotic prescribing pressures may have been reduced given virtual or telephone follow-up. Additionally, at our institution, the infectious disease division played an active role in logistical and resource support for managing ambulatory COVID-19 encounters, likely easing provider concerns pertaining to withholding antibiotics. As antimicrobial stewardship teams look to optimize antimicrobial utilization in COVID-19, our findings suggest that efforts should be focused on the inpatient and ED settings. Attempts to further optimize outpatient antimicrobial prescribing in COVID-19 cases, where ID specialists are already actively involved likely represents a scenario in which effort could far exceed the potential impact.

Our study has several limitations. Most importantly, our center is a large academic center in a relatively small community located in an area of low disease prevalence, which may limit external validity. Laboratory studies testing for the presence of coinfection were not employed in all patients. Thus, coinfection rates reported may not represent the absolute rates that may have been observed had testing been widely utilized. Also, these data were collected from patients seen very early on in the pandemic, when information regarding coinfection with bacterial pathogens was sparse.

Detailed data specific to antimicrobial use in patients with COVID-19 across the care continuum are currently limited. When reported, specific antimicrobial utilization metrics are often missing. A pertinent strength of our study is the application of spectrum scoring in addition to other common antimicrobial use metrics. Most importantly, we have described data pertaining to antimicrobial utilization in 287 patients diagnosed and managed as outpatients. An understanding of antimicrobial prescribing rates across the continuum of care, as reported here, will allow facilities to carefully craft targeted antimicrobial stewardship strategies to contribute to the minimization of unnecessary and/or inappropriate antimicrobials in patients with COVID-19.

At our center, increased prescribing rates and broader spectrums of antimicrobial activity seemed to be used in patients with greater disease severity and those requiring a higher level of care. However, increased antimicrobial utilization did not coincide with increased observation of bacterial coinfection. We are reassured to know that inpatient management of nonsevere cases and outpatient management of mild cases did not involve widespread antimicrobial usage. Management of COVID-19 patients must reflect available and evolving evidence. Bacterial coinfection seems to be rare, even more so in areas of low prevalence. Routine use of broad-spectrum agents and long durations of antimicrobials in patients with COVID-19 are likely unnecessary and could contribute to the epidemic of antimicrobial resistance. Larger studies across multiple institutions and geographic regions are needed to further explore this subject and to define optimal antimicrobial stewardship strategies.

## References

[1] Clancy CJ , Nguyen MH. COVID-19, superinfections and antimicrobial development: what can we expect? Clin Infect Dis 2020 May 1 [Epub ahead of print]. doi: 10.1093/cid/ciaa524. PMC7197597 32361747

[2] Rawson TM , Moore LSP , Zhu N , et al. Bacterial and fungal co-infection in individuals with coronavirus: a rapid review to support COVID-19 antimicrobial prescribing. Clin Infect Dis 2020 May 2 [Epub ahead of print]. doi: 10.1093/cid/ciaa530. PMC7197596 32358954

[3] COVID-19 rapid guideline: antibiotics for pneumonia in adults in hospital. National Institute for Health Care Excellence website. www.nice.org.uk/guidance/ng173 May 2020. Published May 2020. 33400459

[4] Antibiotic/Antimicrobial resistance (AR/AMR). Centers for Disease Control and Prevention website. https://www.cdc.gov/drugresistance/index.html. Published February 2020. Accessed July 23, 2020.

[5] Gerber JS , Hersh AL , Kronman MP , Newland JG , Ross RK , Metjian TA. Development and application of an antibiotic spectrum index for benchmarking antibiotic selection patterns across hospitals. Infect Control Hosp Epidemiol 2017;38:993–997. 28560946 10.1017/ice.2017.94

[6] Arentz M , Yim E , Klaff L , et al. Characteristics and outcomes of 21 critically ill patients with COVID-19 in Washington state. JAMA 2020;323:1612–1614. 10.1001/jama.2020.4326 PMC7082763 32191259

[7] Chen L , Liu HG , Liu W , et al. Analysis of clinical features of 29 patients with 2019 novel coronavirus pneumonia. Zhonghua Jie He He Hu Xi Za Zhi 2020;43:203–208. 32164089 10.3760/cma.j.issn.1001-0939.2020.03.013

[8] Chen N , Zhou M , Dong X , Qu J , Gong F , Han Y , et al. Epidemiological and clinical characteristics of 99 cases of 2019 novel coronavirus pneumonia in Wuhan, China: a descriptive study. Lancet 2020;395:507–513. 32007143 10.1016/S0140-6736(20)30211-7 PMC7135076

[9] Dong X , Cao YY , Lu XX , et al. Eleven faces of coronavirus disease 2019. Allergy 2020;75:1699–1709. 32196678 10.1111/all.14289 PMC7228397

[10] Goyal P , Choi JJ , Pinheiro LC , et al. Clinical characteristics of COVID-19 in New York City. N Engl J Med 2020;382:2372–2374. 32302078 10.1056/NEJMc2010419 PMC7182018

[11] Huang C , Wang Y , Li X , et al. Clinical features of patients infected with 2019 novel coronavirus in Wuhan, China. Lancet 2020;395:497–506. 31986264 10.1016/S0140-6736(20)30183-5 PMC7159299

[12] Yu N , Li W , Kang Q , et al. Clinical features and obstetric and neonatal outcomes of pregnant patients with COVID-19 in Wuhan, China: a retrospective, single-centre, descriptive study. Lancet Infect Dis 2020;20:559–564. 32220284 10.1016/S1473-3099(20)30176-6 PMC7158904

[13] Zhou F , Yu T , Du R , et al. Clinical course and risk factors for mortality of adulte inpatients with COVID-19 in Wuhan, China: a retrospective cohort study. Lancet 2020;395:1054–1062. 32171076 10.1016/S0140-6736(20)30566-3 PMC7270627

[14] Yang K , Sheng Y , Huang C , et al. Clinical characteristics, outcomes, and risk factors for mortality in patients with cancer and COVID-19 in Hubei, China: a multicenter, retrospective, cohort study. Lancet Oncol 2020;21:904–913. 32479787 10.1016/S1470-2045(20)30310-7 PMC7259917

[15] Stenehjem E , Wallin A , Fleming-Dutra KE , et al. Antibiotic prescribing variability in a large urgent-care network: a new target for outpatient stewardship. Clin Infect Dis 2020;70:1781–1787. 31641768 10.1093/cid/ciz910 PMC7768670

[16] Walsh TL , Taffe K , Sacca N , et al. Risk factors for unnecessary antibiotic prescribing for acute respiratory tract infections in primary care. Mayo Clin Proc Innov Qual Outcomes 2020;4(1):31–39. 32055769 10.1016/j.mayocpiqo.2019.09.004 PMC7011009

[17] Dempsey PP , Businger AC , Whaley LE , Gagne JJ , Linder JA. Primary care clinicians’ perceptions about antibiotic prescribing for acute bronchitis: a qualitative study. BMC Fam Pract 2014;15:194. 25495918 10.1186/s12875-014-0194-5 PMC4275949

